# Supplementary material for: Experimental verification of the application of lateral buildup ratio on the 4‐MeV electron beam
Source: J Appl Clin Med Phys. 2006 Feb 21;7(1):35–41. doi: 10.1120/jacmp.v7i1.2153 (PMC5722474; doi:10.1120/jacmp.v7i1.2153)
Supplement: Supplementary file 1 — Supplementary Material Files [file ACM2-7-035-s001.doc]

# Experimental Verification of the Application of Lateral Build-up Ratio on the 4 MeV Electron Beam

James C.L. Chow^1,2,3^ and Scott Newman^1^

^1^Medical Physics Department, Grand River Regional Cancer Center, Grand River Hospital, PO Box 9056, 835 King Street West, Kitchener, Ontario, Canada N2G 1G3

^2^Department of Physics, University of Waterloo, 200 University Avenue, Waterloo,

Ontario, Canada N2L 3G1

# ^3^Permanent Address: Radiation Medicine Program, Princess Margaret Hospital, 610 University Avenue, Toronto, Ontario, Canada M5G 2M9

**Abstract**

The Lateral Build-up Ratio (LBR) used to estimate the depth dose distribution of electron beams for an irregular cutout field was obtained for a 4 MeV energy beam from a Varian 21 EX linear accelerator. The depth dose curves for a group of circular cutout fields starting from a 2 cm diameter were measured. Electron diodes were used in a large water tank, to measure the LBR values for 6, 9, 12 and 16 MeV electron beam energies and a 10 cm x 10 cm applicator. The results agreed with the published data. When the same equipment, setup and technique were used to determine the LBR values for the 4 MeV energy beam, the values were only reasonable, being within the clinical treatment range (i.e. LBR < 1) for the smallest 6 cm x 6 cm applicator. The calculated LBR values were clinically unacceptable for the circular cutout fields with a diameter larger than 2 cm with the 10 cm x 10 cm applicator. The difficulty in the LBR measurement may be due to the significant contribution of scattered electrons from the beam defining system. This study also focused on how well the sigma values for the 4 MeV beam can predict depth dose curves for other field sizes and whether the values are applicator dependent.

## Keywords: electron therapy, dosimetry, lateral build-up ratio

**I. Introduction**

It is well known that the elementary pencil beam algorithms ^(1-5)^ have been used to calculate the dose distribution for electron beam treatment planning. These algorithms required measured beam data of depth doses and beam profiles which varied with the collimation systems of different accelerators. For that, the dose per monitor unit of irregularly shaped cutout fields has individually been measured in clinical practice. This patient specific measurements were time consuming, so different models based on the pencil beam approach were proposed to calculate the output factor in irregularly shaped electron fields.^(6-9)^ Khan *et al* ^(10-12)^ introduced a semiempirical model based on the lateral spread of the pencil beam and proposed a function called the Lateral Build-up Ratio (LBR). It is defined as the ratio of dose at a point of depth for a given circular field to the dose at the same point for a reference broad field, which is large enough to provide the lateral scatter equilibrium for the incident fluence and profile. The LBR represents the fractional change in dose at a field point due to the loss of scatter relative to the large reference field and can be calculated from the percentage depth dose (PDD) normalized near to the surface. The ratio is related to the lateral pencil beam spread function, σ(z, E), where z is the depth and E is the energy of a circular electron beam of radius r.^(11,12)^ By using the σ_r_(z, E) values derived from the measured LBRs for a small circular reference field (usually with a 2 cm diameter), sector-type integration can be used to calculate the average LBR at any depth of an irregular field.^(10,12-14)^

Khan *et al*. have demonstrated the convenience and accuracy of using the LBR to calculate the electron beam output factors for 6, 9, 12, 16 and 20 MeV electron beams,^(10-12)^ and Higgins *et al* ^(15)^ have further evaluated the application of this model to calculate dose outside the field edge and in a heterogeneous media. Although the above five electron beam energies are more frequently used by most centers for clinical treatment, 4 MeV is an available option.^(16)^ This energy, with its relatively shorter practical range and depth of maximum dose (d_m_), is particularly useful for treating superficial lesions when the superficial x-ray treatment unit is not available.^(17-20)^ However, 4 MeV beam data such as depth dose and beam profiles are more unstable, due to difficult beam tuning and are difficult to measure accurately compared to higher energies.^(21-23)^ The aim of this study is to investigate whether the LBR approach is beneficial to the 4 MeV electron beam through measurements.

# II. Materials and Methods

### A. Beam Profiles and Percentage Depth Dose

# The beam profiles and PDD curves of the electron beams were measured using a scanning water tank system (RFA 300, Scanditronix Medical AB with Omni Pro 6 software). A waterproof high-doped p-type silicon diode (Scanditronix Medical AB, EFD-3G) was used to measure both the beam profiles and PDD at the central beam axis. The thickness of the silicon chip was 0.5 mm and the diameter of the active area was 2 mm. Since some of the circular cutout fields were too small to put a reference detector on the beam path, the reference dose signal for the measurements was obtained from the internal monitoring ionization chamber within the gantry head. A Varian 21 EX linear accelerator (linac) with 4, 6, 9, 12 and 16 MeV clinical electron beams, was used in the measurement. The 4 MeV electron beam uses a scattering foil designed for the specific energy. The central axis for the PDD curve was located according to the peak position of the profile for the measurement. This was particularly important when performing the PDD measurement for a very small circular cutout field (close to 2 cm diameter), where the beam penumbras were relatively large for such a low energy due to electronic disequilibrium.

The diode was positioned vertically, perpendicular to the water surface. This setting made the sampling resolution dependent on the thickness of the diode sensitive volume (~ 0.5 mm). The position of the sensitive region from the detector front surface was provided by the manufacturer and verified in this study, and was considered the effective point of measurement. A depth ionization curve was scanned first to determine the position of d_m_ before the beam profile scanning. The sensitive volume of the diode was then positioned there and the beam profiles along the in- and cross-plane directions were scanned. Both the 6 cm x 6 cm and 10 cm x 10 cm applicators were used in the measurement. For the 10 cm x 10 cm applicator, circular cutouts with diameters of 2, 3, 4, 6, 8 and 10 cm and square cutout of 10 x 10 cm^2^ were made. While for the 6 cm x 6 cm applicator, circular cutouts with diameters of 2, 2.5, 3, 4, 5 and 6 cm and square cutout of 6 cm x 6 cm were made. The thickness of cutout in this study was 15 ± 1mm and the field edges were shape and not divergent. The beam profiles for all cutouts of the 10 cm x 10 cm applicator with 4, 6, 9, 12 and 16 MeV were measured. While for the cutouts of the 6 cm x 6 cm applicator, only the 4 MeV beam profiles were measured.

The position of the surface was determined by noting the dose variation in the diode reading at the water-air interface. Percentage Depth Ionization (PDI) curves were measured with the highest sampling resolution and the slowest speed. Central beam axis PDI curves for the different cutouts of the 10 cm x 10 cm applicator as mentioned above were measured for all five energies. For the cutouts of the 6 cm x 6 cm applicator, only the 4 MeV PDI curves were measured. All measurements were taken using a Source to Surface Distance (SSD) of 100 cm with an air gap of 5 cm. These measurements were carefully repeated one by one within the same day. It was found that the repeated scan agreed with the original results within ± 0.5 %. The SSD and zero-water-level were checked frequently in order to prevent any physical effects, such as evaporation, from introducing measurement error. In addition, the actual cutout dimensions used for the measurements were checked to ensure the center of the circular cutout was positioned at the central beam axis within ± 2 mm. The radiation characteristics of the diode were verified with the ionization chamber to confirm that the depth ionization curve obtained by the diode could be used as the depth dose curve without correction.

# B. Formalism and LBR Calculation

The LBR is defined as ^(10)^

$LBR (r,z,E) = \frac{D(r,z,E)}{D(r_{\infty},z,E)} \cdot\frac{\Phi(r_{\infty},E)}{\Phi(r,E)} ,$ (1)

where D is the dose, r is the radius of the field defined at the water surface (in our study, it was SSD = 100 cm), z is the depth, E is the incident electron beam energy. Φ is the incident fluence and r_∞_ is the broad field radius. That is the radius of field large enough to provide lateral scatter equilibrium. In this study, the variation of the incident fluence factor in Equation (1) was factored out by normalizing the depth dose data of the circular field to the dose near to the surface. The broad fields for the 6 cm x 6 cm and 10 cm x 10 cm applicator were selected to be the open square cutout fields of 6 cm x 6 cm and 10 cm x 10 cm respectively.

Since LBR can be written as

$LBR (R,z) = 1 - \exp(\frac{-r^{2}}{\sigma_{r}^{2}(z)}) ,$ (2)

and therefore

$\sigma_{r}(z) = \frac{r}{\sqrt{\ln\left[\frac{1}{1-LBR(r,z)}\right]}}$, (3)

provided that LBR value is smaller than 1, it is also possible to determine the σ_r_(z) values with respect to the depth, z, or normalized depth, z/R_p_, for each electron beam energy.

**III. Results**

Figure 1 is a plot of σ_r_ as a function of the normalized depth for the 4, 6, 9, 12 and 16 MeV energies. The values were calculated using the LBR data for the 2 cm diameter circular cutout as suggested by Khan *et al*.^(10)^ According to the ICRU Report 35 (ICRU 1984),^(24)^ measuring the dose at a depth of 0.5 mm instead of at the surface was suggested as a normalization to ensure proper detector positioning and to avoid measurements in the unstable build-up region near the surface. Figures 2(a) and 2(b) show the 4 MeV PDD curves using the circular cutouts with 2, 2.5, 3, 4, 5 and 6 cm diameters for the 6 cm x 6 cm and 10 cm x 10 cm applicators respectively. The PDD curves were normalized to their doses at 0.5 mm from the water surface. The LBR curves against the normalized depth, z/R_p_, calculated from Figs. 2(a) and 2(b) are shown in Figs. 3(a) and 3(b) respectively. All LBR values larger than one were eliminated in Figs. 3.

**IV. Discussion**

In Fig. 1, the σ_r_ values were calculated using the 2 cm diameter cutout for the 10 cm x 10 cm applicator. These values were verified and agreed with the similar published results ^(10)^ except for the 4 MeV energy. It was found that, as previously reported,^(12)^ the PDD calculated using our σ_r_ values can reproduce the PDD for other field sizes with energies from 6 to 16 MeV within ± 1 % error bar. While for the 4 MeV, the error in reproducing the PDD is ± 4 %. Moreover, the σ_r_ vs. z/R_p_ and LBR vs. z/R_p_ are independent of the cutout shape as reported.^(10)^ There is no problem in the application of LBR in the above energy range.

In Fig. 2(a) showing the PDD curves of the 6 cm x 6 cm applicator, it was found that beyond 1.5 cm, the depth doses of the circular cutouts with diameters equal to or larger than 5 cm are very close or even very slightly larger (about 3 % on average) than that of the broad field. Such uncertainty is larger than the reproducibility error bar of the measurement (± 0.5 %). In Fig. 2(b), there was a more significant increase in the depth doses of cutouts (> 5 cm) than in the broad field, in the bremsstrahlung tail range for the 10 cm x 10 cm applicator. In the two figures, when the electronic equilibrium condition had been reached for those sufficiently large circular fields (i.e. > 5 cm diameter), measurement uncertainty of the depth dose near to the bremsstrahlung tail became significant in the LBR calculation. Or, it is understood that, those large circular fields can also be recognized as broad fields. For the 4 MeV energy, neglecting the consideration for the physical effects from the measurement, the PDD curve is very difficult to predict through measurements, especially for depth larger than 2 cm, because the scattered electrons from the applicator and cutout contribute significantly to the doses, due to its relatively large electron angular scattering cross section. Such dose contribution from the scattered electrons is difficult to predict because it depends on the material and geometry of the beam defining system. The scattered electrons from the applicator have a different energy spectrum and angular scattering cross section than the primary incident electrons. The difference of field size dependence between the 6 cm x 6 cm and 10 cm x 10 cm applicator is also probably due to the different beam defining system used, though both applicators have the same photon jaws setting (20 cm x 20 cm). For highly accurate results, Monte Carlo investigations would be useful but are beyond the scope of this study.

Figures 3 show the LBR values calculated from Figs. 2. In Fig. 3(a), the LBR values are seen to be nonsense beyond 15 mm or normalized depth (z/R_p_) of 0.88. However, the values are reasonable if the focus is restricted to the clinical treatment range between 7 mm (d_m_) and 14 mm (i.e. 100 to 50% isodose contour), or normalized depth between 0.41 and 0.82, and excludes the LBR calculated from the large circular cutouts equivalent to the broad field. The discontinuities near R_p_ in Fig. 3(a) are due to the measured depth dose uncertainty as explained in Fig. 2(a). However, in Fig. 3(b) for the 10 cm x 10 cm applicator, it is seen that only the LBR values of circular cutouts with 2 cm and 3 cm diameter are reasonable. It seems that the LBR was hard to measure and calculate for the 4 MeV electron energy, using an applicator larger than 6 cm x 6 cm within the clinical treatment range. In the discontinuity range of the figures, the sigma values calculated using the PDD data of the 2 cm cutout could not predict the PDD data for other field sizes accurately. This is different for higher energies from 6 to 16 MeV, where the PDD calculated using sigma values for a 2 cm diameter cutout could reproduce the PDD for other field sizes as reported.^(10)^ As the PDD data for higher energies is applicator independent, according to our measurement, the sigma values and LBR vs z/R_p_ is also applicator independent. It has been verified for the 6 MeV electron beam. However, this is not the case for the low energy 4 MeV beam, as shown in Figs. 2 and 3. Different LBR vs z/R_p_ should be plotted for different applicators.

**V. Conclusion**

The LBR values of the 4 MeV electron energy using the Varian 21 EX accelerator were measured and calculated. It was found that, for a small applicator size of 6 cm x 6 cm, the calculated LBR values were reasonable and within the clinical treatment range. However, when a larger 10 cm x 10 cm applicator was used, the LBR values with circular fields larger than 2 cm in diameter were not reasonable compared to those of higher energies. This is because the PDD for a range of cutouts was higher than the 10 cm x 10 cm applicator broad beam PDD at certain depth ranges, and the LBR model breaks down under these conditions. It was found that the depth dose uncertainty increased when an insert and applicator larger than 6 cm x 6 cm was used. Another reason is that the lateral scatter/electronic equilibrium is more easily reached by increasing the circular cutout size from 2 cm diameter compared to higher energies. Since, in the output (dose per monitor unit), calculation of an irregular field requires sector-type integration of LBR with a variation of radii data, the uncertain LBR values in the larger circular fields cause significant error in the output estimation of the 4 MeV electron beam compared to those at higher energies. Based on the measured results in this study, it can be concluded that the LBR could not be used to model the 4 MeV electron beam well.

**Reference**

^1^ Perry DJ and Holt JG. A model for calculating the effects of small inhomogeneities on electron beam dose distribution. Med. Phys. 1980; **7**: 207-215.

^2^ Brahme A, Lax I and Andreo P. Electron beam dose planning using discrete Gaussian beams, mathematical background. Acta. Radiol. Oncol. 1981; **20**: 147-158.

^3^ Hogstrom KR, Mills MD and Almond PR. Electon beam dose calculations. Phys. Med. Biol. 1981; **26**: 445-459.

^4^ Werner BL, Khan FM and Deibel FC. A model for calculating electron beam scattering in treatment planning. Med. Phys. 1982; **9**: 180-187.

^5^ Jette D. The application of multiple scattering theory to therapeutic electron dosimetry. Med. Phys. 1983; **10**: 141-146.

^6^ Dutreix A and Briot E. The development of a pencil-beam algorithm for clinical use at the Institut Gustave Roussy. in *The Computation of Dose Distributions in Electron Beams,* Radiotherapy, edited by A. E. Nahum (University of Umea, Sweden, 1990), pp. 242-270.

^7^ Mills MD, Hogstrom KR and Almond PR. Prediction of electron beam output factors. Med. Phys. 1982; **9**: 60-68.

^8^ Bruinvis IAD, Amstel AV, Elevelt AJ and Van der Laarse R. Dose calculation for arbitrarily shaped electron fields. Acta Radiol. Suppl. 1983: **364**: 73-79.

^9^ McParland BJ. A method of calculating the output factors of arbitrarily shaped electron fields. Med. Phys. 1989; **16**: 88-93.

^10^ Khan FM, Higgins PD, Gerbi BJ, Deibel FC, Sethi A and Mihailidis DN. Calculation of depth dose and dose per monitor unit for irregularly shaped electron field. Phys. Med. Biol. 1998; **43**: 2741-2754.

^11^ Khan FM and Higgins PD. Calculation of depth dose and dose per monitor unit for irregularly shaped electron fields: an addendum. Phys. Med. Biol. 1999; **44**: N77-N80.

^12^ Khan FM and Higgins PD. Field equivalence for clinical electron beams. Phys. Med. Biol. 2001; **46**: N9-N14.

^13^ Jones D, Andre P, Washington JT, and Hafermann HD. A method for the assessment of the output of irregularly shaped electron fields. Br. J. Radiol. 1990; **63**: 59-64.

^14^ Wu RK, Wang W and El-Mahdi AM. Irregular field output factors for electron beams. in *Proceedings of the 9^th^ International Conference on the Use of Computers in Radiation therapy*, edited by I.A.D. Bruvinvis, P.H. van der Giessen, H.J. van Kleffens, and F.W. Wittkamper (Scheveningen, Sweden, 1987), pp. 453-456.

^15^ Higgins PD, Gerbi PD and Khan FM. Application of measured pencil beam parameters for electron beam model evaluation. Med. Phys. 2003; **30**: 514-520.

^16^ Followill DS, Davis DS and Ibbott GS. Comparison of electron beam characteristic from multiple accelerators. Int J Radiat Oncol Biol Phys. 2004; **59**: 905-910.

^17^ Walker C, Wadd NJ and Lucraft HH. Novel solutions to the problems encountered in electron irradiation to the surface of the head. Br J Radiol. 1999; **72**: 787-791.

^18^ Scholten AN, Griep C, Davelaar J, Chin A and Leer JW. Electron beam irradiation is effective in the treatment of skin carcinomas; a comparison with superficial roentgen therapy. Ned Tijdschr Geneeskd. 1996; **148**: 428-431.

^19^ Amdur RJ, Kalbaugh KJ, Ewald LM, Parsons JT, Mendenhall WM, Bova FJ and Million RR. Radiation therapy for skin cancer near the eye: kilovoltage x-rays versus electrons. Int J Radiat Oncol Biol Phys. 1992; **23**: 769-779.

^20^ Das IJ, Kase KR, Copeland JF and Fitzgerald TJ. Electron beam modifications for the treatment of superifical malignancies. Int J Radiat Oncol Biol Phys. 1991; **21**: 1627-1634.

^21^ van Battum LJ and Huizenga H. On the initial angular variances of clinical electron beams. Phys. Med. Biol. 1999: **44**: 2803-2820.

^22^ McParland BJ, Cunningham JR and Woo MK. The optimization of pencil beam widths for use in an electron pencil beam algorithm. Med. Phys. 1988; **15**: 489-497.

^23^ Li XA and Rogers DWO. Electron mass scattering powers: Monte Carlo and analytical calculations. Med. Phys. 1995; **22**: 531-541.

^24^ Radiation dosimetry: electron beams with energy between 1 and 50 MeV. ICRU Report 35 1984; Bethesda, MD: ICRU.

**Figure Captions**

1 σ_r_ plotted against the normalized depth, z/R_p_, for the 4 - 16 MeV electron beam. The values were calculated from the LBR data for the 2 cm diameter field for the 10 cm x 10 cm applicator.

2(a) 4 MeV PDD curves of circular cutouts with diameters 2, 2.5, 3, 4, 5, 6 cm for (a) 6 cm x 6 cm applicator and (b) 10 cm x 10 cm applicator. All curves are normalized to the dose near to the water surface (0.5 mm depth of water).

3 LBR curves calculated from (a) Fig. 2(a), and (b) Fig. 2(b) respectively. The curves are plotted against the depth normalized to the practical range, R_p_, of the 4 MeV beam.
